# Oral paracoccidioidomycosis: a retrospective study of 95 cases from a single center and literature review

**DOI:** 10.4317/medoral.25613

**Published:** 2023-02-18

**Authors:** Lucineide Lima Cardoso de de Oliveira, José Alcides Almeida de de Arruda, Marcia Frias Pinto Marinho, Israel Leal Cavalcante, Lucas Guimarães Abreu, Aline Corrêa Abrahão, Mario José Romañach, Bruno Augusto Benevenuto de de Andrade, Michelle Agostini

**Affiliations:** 1 Department of Oral Diagnosis and Pathology, School of Dentistry, Universidade Federal do Rio de Janeiro, Rio de Janeiro, Brazil; 2 Department of Oral Surgery, Pathology and Clinical Dentistry, School of Dentistry, Universidade Federal de Minas Gerais, Belo Horizonte, Minas Gerais, Brazil; 3 Department of Oral Pathology, School of Dentistry, Universidade de Fortaleza, Fortaleza, Ceará, Brazil; 4 Department of Child and Adolescent Oral Health, School of Dentistry, Universidade Federal de Minas Gerais, Belo Horizonte, Minas Gerais, Brazil

## Abstract

**Background:**

The ecoepidemiological panorama of paracoccidioidomycosis (PCM) is dynamic and still ongoing in Brazil. In particular, data about the oral lesions of PCM are barely explored. The aim of this study was to report the clinicopathological features of individuals diagnosed with oral PCM lesions at an oral and maxillofacial pathology service in Rio de Janeiro, Brazil, in the light of a literature review.

**Material and Methods:**

A retrospective study was conducted on oral biopsies obtained from 1958 to 2021. Additionally, electronic searches were conducted in PubMed, Embase, Scopus, Web of Science, Latin American and Caribbean Center on Health Sciences Information, and Brazilian Library of Dentistry to gather information from large case series of oral PCM.

**Results:**

Ninety-five cases of oral PCM were surveyed. The manifestations were more frequent among males (*n*=86/90.5%), middle-aged/older adults (*n*=54/58.7%), and white individuals (*n*=40/51.9%). The most commonly affected sites were the gingiva/alveolar ridge (*n*=40/23.4%) and lip/labial commissure (*n*=33/19.3%); however, one (*n*=40/42.1%) or multiple sites (*n*=55/57.9%) could also be affected. In 90 (94.7%) patients, “mulberry-like” ulcerations/moriform appearance were observed. Data from 21 studies (1,333 cases), mostly Brazilian (90.5%), revealed that men (92.4%; male/female: 11.8:1) and individuals in the fifth and sixth decades of life were the most affected (range: 7-89 years), with the gingiva/alveolar ridge, palate, and lips/labial commissure being the sites most frequently affected.

**Conclusions:**

The features of oral PCM lesions are similar to those reported in previous studies from Latin America. Clinicians should be aware of the oral manifestations of PCM, with emphasis on the clinicodemographic aspects and differential diagnoses, especially considering the phenomenon of the emergence of reported cases in rural and/or urban areas of Brazil.

** Key words:**Fungal infection, mycoses, neglected diseases, oral manifestations, oral medicine, oral mucosa, paracoccidioidomycosis, south american blastomycosis.

## Introduction

Paracoccidioidomycosis (PCM), previously identified as South American blastomycosis, is caused by the human fungal pathogens of the *Paracoccidioides genus* (1). Though a rare disease worldwide, PCM is the second most common endemic deep mycosis in Latin America and the first in Brazil (1-3), having been recently acknowledged as a neglected tropical (fungal) disease by the World Health Organization and the Pan American Health Organization (3). According to current estimates, 10 million people in Latin America are infected with PCM, which, in turn, represents the main cause of death attributed to systemic mycoses (1,2,4). However, the real burden of PCM may be underestimated due to the lack of compulsory notification of cases. In Brazil, for example, PCM was recently included in the National List of Compulsory Notification of diseases, injuries, and public-health events, but few states have included this fungal disease in their local records of public health-reporTable diseases (2,4).

Changes in the ecoepidemiological aspects of PCM have been noticed in Brazil in recent decades due to the advent of new agricultural practices (1,5). Soil is the main natural source of infectious conidia and people who live in rural regions and manage the soil directly or indirectly represent a risk group for PCM (1,4). Both animals and humans can acquire PCM by inhalation (1). According to the incubation period and characteristics of affected individuals, PCM usually causes a transient lung infection that can progress to an acute/subacute form or, more often, can reactivate later as a chronic and insidious disease (1,4). In addition to the lungs, upper airway tract, lymph nodes, skin, adrenal glands, central nervous system, and digestive tract (including oral cavity) can also be affected (1,2,4-7).

Oral mucosal lesions may be the first noticeable physical manifestation of PMC since about 60% of individuals diagnosed with the disease may exhibit oral and/or oropharyngeal involvement (8). Nevertheless, the diagnosis of PCM through oral lesions can be challenging due to similarity with other conditions such as other fungal diseases, protozoal and bacterial infections, as well as malignant neoplasms (6,7). In a large Brazilian multicenter study, this lesion represented 0.3% of the specimens of oral biopsies submitted to histopathological evaluation (7). Knowledge about the trends and social impact of oral PCM in regions of high endemicity, including the state of Rio de Janeiro in Brazil, may still be limited. In addition, most of the available data remain diluted among other clinical features of PCM and may have been unnoticed in the reports or may have come from single case reports or case series (6-14). Thus, examining the clinicodemographic data of oral PCM from multiple databases may be useful to understand the current scenario of the disease and to state future directions that might been taken on this issue.

Herein, we report 95 additional cases of oral PCM diagnosed at a referral service of oral and maxillofacial pathology in Rio de Janeiro, Brazil. The study also provides an overview of clinicodemographic aspects and the management of the condition, based on large case series of oral PCM reported in the literature.

## Material and Methods

- Case series

The cases of oral PMC diagnosed at the Oral Pathology Laboratory of the School of Dentistry, Federal University of Rio de Janeiro were obtained by reviewing the Institution's files for the period between 1958 and 2021. The study was approved by the Ethics Committee of the local Institution (No. 54005021.7.0000.5257). The patient's identity remained anonymous according to the ethical principles of the Declaration of Helsinki.

The following data were collected from the records of individuals: sex, age, skin color, occupation, time of evolution of the lesion, symptomatology, clinical aspects, anatomical location, and clinical differential diagnosis. For anatomical location and differential diagnosis, the unit of analysis was not the number of individuals, since each individual evaluated could have been affected at more than one anatomical site and the clinician may have formulated more than one diagnostic hypothesis. All cases were referred by dentists.

All cases of oral PCM from the period under study were included, and those with incomplete data were excluded. After selection of the cases, 5-mm thick sections were cut from the paraffin blocks, stained with hematoxylin-eosin (H&E), and re-examined under light microscopy by two lecturers of Oral and Maxillofacial Pathology (B.A.B.A.; M.J.R.) for diagnostic confirmation. Yeasts were identified after staining the tissue sections with the Grocott-Gomori methenamine silver.

- Literature review

Electronic searches were conducted in PubMed, Embase, Scopus, Web of Science, Latin American and Caribbean Center on Health Sciences Information (LILACS), and Brazilian Library of Dentistry (BBO) in February 21, 2022 and updated in July 6, 2022. The following combination of terms was used: (paracoccidioidomycosis OR “South American blastomycosis” OR “paracoccidioidal granuloma” OR “Lobo disease” OR “Lutz-Splendore-Almeida disease”) AND (“alveolar process” OR “alveolar ridge” OR “buccal mucosa” OR “buccal mucosal” OR “floor of the mouth” OR gingiva OR gingivae OR “hard palate” OR jaw OR jaws OR lip OR lips OR mandible OR mandibles OR maxilla OR maxillae OR mouth OR oral OR “oral cavity” OR “oral mucosa” OR “oral mucosae” OR oropharynges OR oropharynx OR palate OR perioral OR “soft palate” OR tongue OR tonsil OR tonsils). References that were duplicated across databases were found and eliminated with a command of the EndNote software (End Note® Online, Clarivate Analytics, Canada).

Inclusion criteria were retrospective studies and case series in which at least 10 cases of oral PCM had been included, without restriction of year of publication, language, or geographical region. Exclusion criteria were histopathological, immunohistochemical or molecular studies, *in vitro* studies (e.g., microbiological assays), and letters to the editor/comments/expert opinions, unless any of these types of publication provided sufficient and detailed clinicodemographic aspects about oral PCM cases.

An author (J.A.A.A.) read the included articles and extracted all data from the studies. A second author (B.A.B.A.) double-checked these data. If the authors disagreed, they discussed until the disagreement was resolved. For unresolved cases, another author (L.G.A.) was consulted. The following data were extracted from the articles included in the literature review: author(s) and year of publication, country, number of cases reported, individuals’ sex and age, anatomical location, evolution time, and treatment.

- Data analysis

Data were tabulated in Microsoft Office Excel 2019 (Microsoft® software, Redmond, WA, USA) and analyzed descriptively using GraphPad Prism version 8.0.0 for Windows (GraphPad software, San Diego, CA, USA).

## Results

- Case series

**Figure 1:**
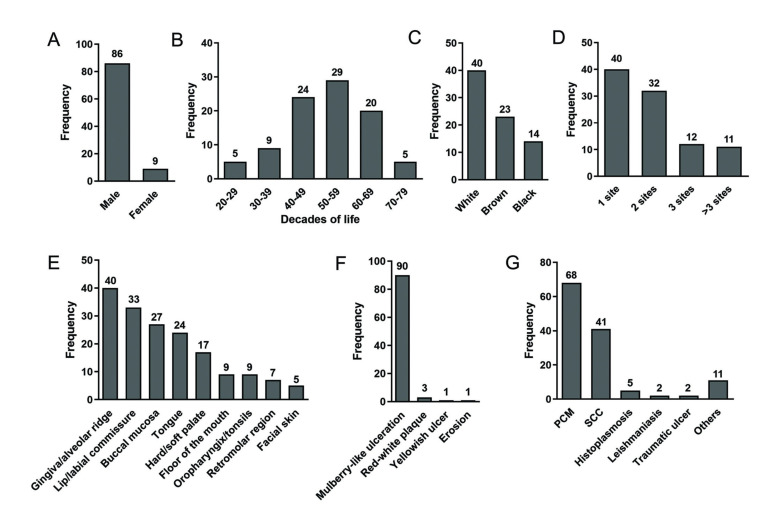
Frequency of oral paracoccidioidomycosis (PCM) cases by (A) sex, (B) decade of life, (C) skin color, (D) number of affected sites, (E) anatomical location, (F) clinical aspects of the lesion, and (G) differential diagnosis provided by clinicians. Notes: SCC, squamous cell carcinoma. The unit of analysis of the variables anatomical location and differential diagnosis was not the number of individuals, but the number of lesions presented and the list possible diagnoses, respectively.

In this 63-year retrospective analysis, 95 cases of oral PCM were identified, representing 0.3% (*n*=25,095) of the histopathological records of oral and maxillofacial biopsies at the service. The clinicodemographic data of the affected individuals are summarized in Fig. 1.

Eighty-six (90.5%) cases occurred in males and nine (9.5%) in females (male-to-female ratio: 9.5:1). Middle-aged and older adults (≥50 years) accounted for 58.7% (*n*=54) of the sample, and the age group most represented was 50-59 years (31.5%). Overall mean age was 51.3±12.0 years (range: 22-78 years); the mean age of the men was 51.6±10.9 years (range: 24-78 years) and the mean age of the women was 47.9±19.0 years (range: 22-77 years). In three patients, information on age was unavailable. White individuals (*n*=40/51.9%) were more affected than non-white individuals [i.e., browns/mulattos (*n*=23/29.9%) and blacks (*n*=14/18.2%)]. Regarding the occupation of affected individuals (*n*=66/95), 21 worked as farmers, followed by retirees (*n*=9), bricklayers/construction workers (*n*=8), salespeople/self-employed (*n*=6), homemakers (*n*=5), students (*n*=2), drivers (*n*=3), carpenters (*n*=3), and *n*=1 for each of the following professions: cook, engineer, physician, packer, plumber, polisher, painter, teacher, and cork producer.

**Figure 2:**
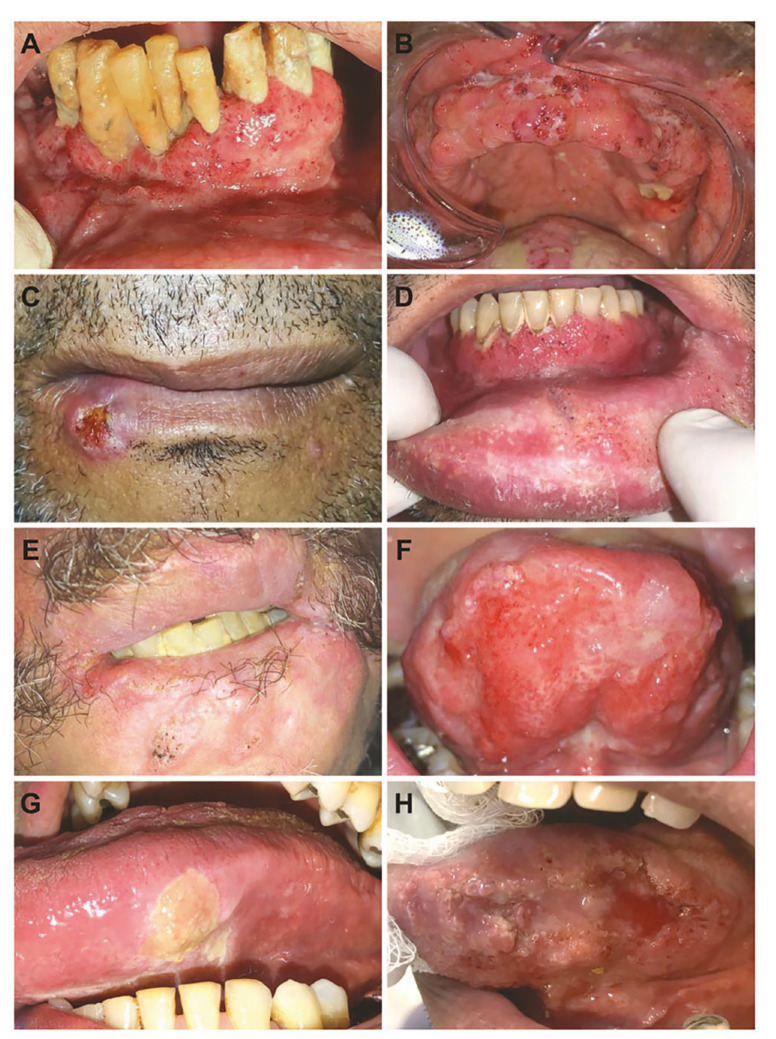
Clinical aspects of oral paracoccidioidomycosis. (A) Mulberry-like ulcers with hemorrhagic dots in the anterior lower gingiva. Poor oral hygiene and associated severe periodontitis are observed. (B) Multiple granular, erythematous, and ulcerated lesions involving the upper alveolar ridge, labial vestibule, and mid-dorsal region of the tongue. (C) Crusted, ulcerated nodule with indurated borders located in the transition between the vermilion of the lip and the outer edge of the lower lip. (D) Moriform appearance and hemorrhagic dots in the anterior lower gingival region. The granular appearance is also seen in both the vestibule and labial mucosa. (E) Irregular, indurated, granular lesion involving the labial commissure and vermilion of the lower lip, as well as the adjacent skin. Note that the lip contour is lost. (F) Diffuse reddish lesion with classic Mulberry-like appearance involving the ventral surface of the tongue. (G) In the border of the tongue, note a yellowish ulcer with ill-defined contours. (H) Exuberant, ulcerated, reddish-white granular lesion on the lateral border of the tongue. The rolled anteroposterior margin was indurated on palpation.

**Figure 3:**
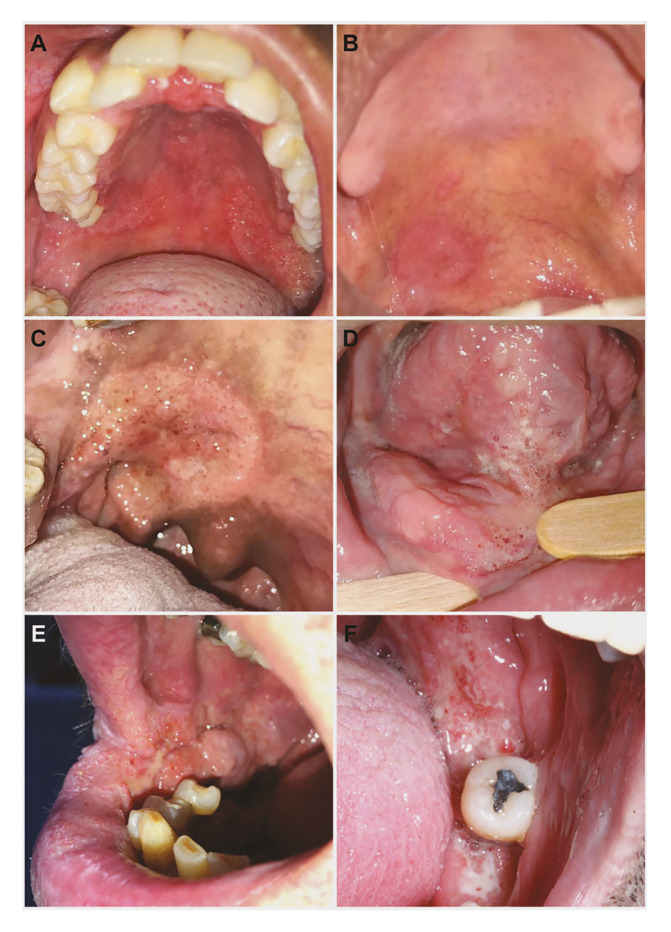
Clinical aspects of oral paracoccidioidomycosis. (A) Moriform appearance: granular and erythematous lesion contoured by irregular edges in the hard and soft palate. (B) Single, rounded, and plaque-like erythematous lesion with regular edges in the soft palate. (C) In the soft palate and tonsil pillar, note an irregular and granular lesion with haemorrhagic dots. (D) On the ventral surface of the tongue, floor of the mouth, and lower alveolar ridge, note a granular, ulcerated and erythematous appearance with yellowish dots. (E) Moriform appearance with irregular and exophytic nodules in the right buccal mucosa. Note that the lesion extends to the vestibule, labial commissure, and vermilion of the lower lip. (F) In the lower alveolar ridge and retromolar trigone, note an erythematous and ulcerated granular lesion with yellowish dots.

The duration of the lesions (*n*=29/95) ranged from 15 days to 24 months, with a median of three months. Regarding symptomatology (*n*=38/95), 26 were symptomatic and 12 were asymptomatic. In 40 cases, one (42.1%) anatomical site was affected, followed by 32 cases affected at two (33.7%) sites, 12 (12.6%) at three sites, and 11 (11.6%) at four or more sites. The gingiva/alveolar ridge (*n*=40/23.4%) was the most commonly affected site, followed by lip/labial commissure (*n*=33/9.3%), and buccal mucosa (*n*=27/15.8%). Clinically, the lesions were mainly “mulberry-like” ulcerations/moriform appearance, i.e., granular, erythematous, and ulcerated lesions (*n*=90/94.7%) (Fig. 2, Fig. 3).

**Figure 4:**
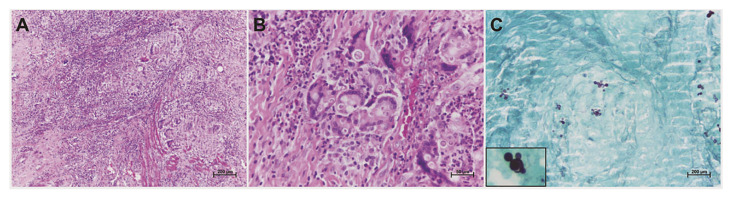
Histopathological features of oral paracoccidioidomycosis. (A) The oral mucosa revealed a chronic granulomatous inflammatory infiltrate in connective tissue. (B) Multinucleated giant cells exhibit numerous rounded structures surrounded by a clear halo (birefringent) in the cytoplasm representing yeast cells of <italic>Paracoccidioides brasiliensis</italic>. (C) Grocott-Gomori staining illustrating the appearance of budding yeasts of <italic>P. brasiliensis</italic> with characteristics of “mariner’s well” or “Mickey Mouse ears” (inset) (hematoxylin and eosin staining: a, ×10 magnification and b, ×40 magnification; Grocott-Gomori staining: c, ×10 magnification).

The most frequent diagnostic hypotheses made by clinicians were fungal and protozoal diseases, with PCM (*n*=68/52.7%) ranking first, followed by squamous cell carcinoma (SCC) (*n*=41/31.8%). Histological sections revealed pseudoepitheliomatous hyperplasia in addition to ulceration of the overlying epithelium with a marked chronic inflammatory infiltrate and multinucleated giant cells detected in the lamina propria. The identification of P. brasiliensis, described as resembling “Mickey Mouse ears” or the spokes of a ship’s steering wheel (“mariner’s wheel”) was observed using Grocott-Gomori staining (Fig. 4).

- Literature review

The electronic searches yielded 1,074 references (PubMed: 231, Embase: 239, Scopus: 392, Web of Science: 163, LILACS: 38, and BBO: 11) and 21 articles met the eligibility criteria (Table 1). With the exception of an article from Argentina and another from Venezuela, the other 19 studies were from Brazil. A total of 1,333 cases of oral PCM were analyzed. Overall, the mean/median of affected individuals were in the fifth (*n*=8 studies/57.2%) and sixth (*n*=5 studies/35.7%) decades of life (range: 7-89 years). Men were the most affected (*n*=1,200; 92.2%), with a male-to-female ratio of 11.8:1. In 31 cases, information on individual’s sex was unavailable. The evolution time of the lesions ranged from 21 days to 72 months. Regarding the anatomical site, the articles provided the percentages of the number of cases. Gingiva/alveolar ridge, palate, and lips/labial commissure were the most frequent sites. Data about patient management were provided in eight studies. The following drugs were used, each alone or in combination: systemic amphotericin B, sulfamethoxazole-trimethoprim, ketoconazole, cotrimoxazole, itraconazole, fluconazole, and sulphadiazine.

**Table 1:**
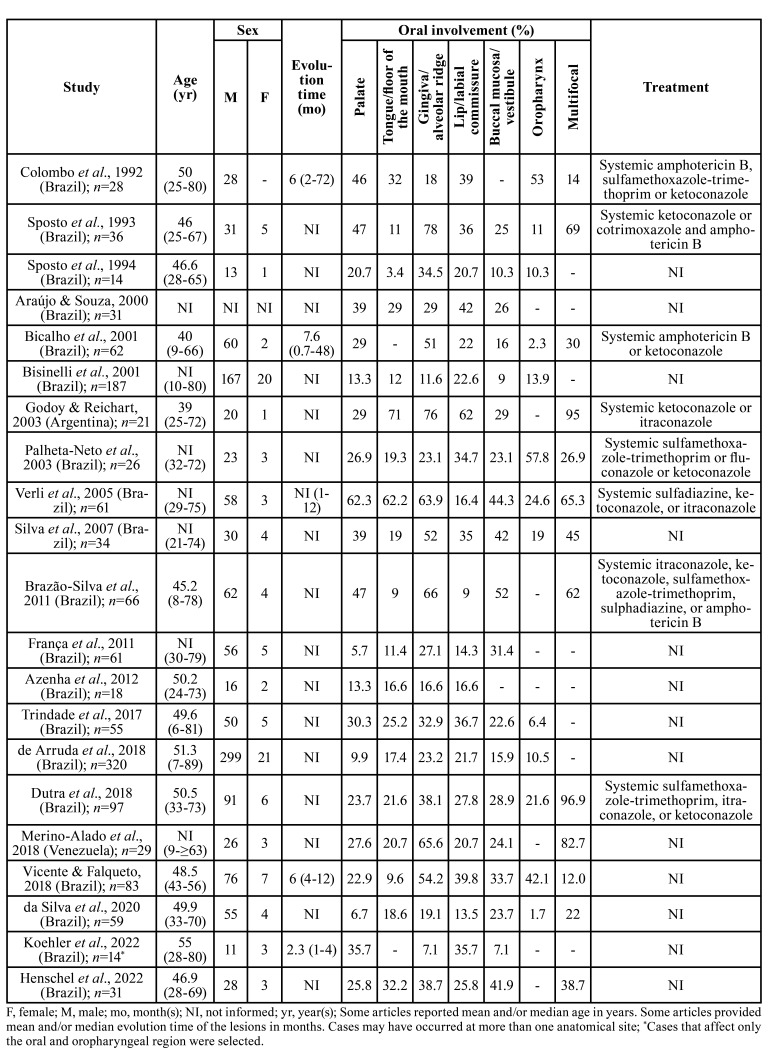
Literature review of articles with large case series of oral paracoccidioidomycosis.

## Discussion

In line with previous single-center or multicenter studies (6,7), in our retrospective assessment oral PCM represented 0.3% of all samples of oral biopsies submitted to histopathological analysis. Compared to the period between 1958 and 1999 (31.6%), the proportional frequency of new diagnosed cases of oral PCM has markedly increased within the last two decades (68.4%), particularly between 2020 and 2021, when 16 cases were diagnosed.

In general, the organs most affected by chronic forms of PCM are the lungs, lymph nodes, and skin. Nevertheless, it is noteworthy that in large PCM registries from Mexico, Brazil, and Venezuela, the occurrence of PCM in the oral cavity ranges from 29.3% to 66%, although the topographic locations were not detailed (15-17). Additionally, previous studies have demonstrated that about 80% of patients exhibited the first manifestations in the oral cavity (6,18). While in our study 100% of oral PCM cases were referred by dentists, Verli *et al*. (19) reported that 57.4% and 29.5% of cases from their oral diagnostic service had been referred by physicians and dentists, respectively. However, some aspects deserve attention. First, although the diagnosis of PCM is routinely based on clinical, radiographic, and microscopy data (1,20), some individuals may be diagnosed by noninvasive tests such as serology (21), even though the latter is not easily available in some South American countries (20). Likewise, other methods used are direct smear, fungal culture, as well as oral and/or pulmonary cytology (1,20). Thus, lesions in the oral cavity are common findings in PCM, but do not appear to be frequently biopsied. In Brazil, this may occur because patients with suspected PCM are referred to tertiary care, mainly due to the involvement of several organs and tissues.

Data from the studies covered by the present comprehensive literature review revealed that Brazil ranked first in absolute number of individuals with oral PCM. Autochthonous cases are exclusive to the tropical and subtropical zone from Mexico (23° north) to Argentina (35° south), being more common in regions with consistent rainfall and at risk from flooding (3). However, the effects of climatic and anthropogenic changes may represent one of the factors linked to the emergence of new cases of oral PCM in non-endemic regions. As such, in a recent series of oral PCM, five of the six individuals studied resided in Northeast Brazil (a non-endemic region) and had not visited any autochthonous region of the country (14). Interestingly, in Europe the disease is a concern since a systematic review has recently revealed 83 cases of PCM diagnosed and reported on that continent (22). The authors have also highlighted the challenges of considering systemic mycosis in the differential diagnosis of individuals who return to their country of origin or immigrate from endemic regions of Latin America (22).

Herein, most cases were from the state of Rio de Janeiro, with the exception of 18, which were from neighboring states/regions. The Southeast region of Brazil encompasses the states of Rio de Janeiro, São Paulo, Minas Gerais, and Espírito Santo, which are historically important zones of high endemicity for the disease (1). The state of Rio de Janeiro has the third highest number of hospitalizations due to PCM in Brazil (23). In this respect, it is important to point out that a rural lifestyle and agricultural activity are very common in this region, with some areas involving abundant cultivation of sugarcane and coffee. More recently, some places have also suffered deforestation both for agriculture and livestock production (5). In addition, the disease has been found to be highly endemic both in metropolitan and mountainous regions (5). In the case of oral PCM in Brazil, data from the literature review revealed that the states of Minas Gerais and Rio Grande do Sul are the ones where most cases have been published, followed by the states of Paraná, Espírito Santo, and São Paulo. In Argentina, cases have also been reported, but in small numbers (12). In our study, 21 infected individuals were farmers; nonetheless, these aspects should be interpreted with caution since patients with PCM usually become infected early in life. Information about the evolution time of the oral lesion was provided for approximately one-third of the patients, with a median of three months. Moreover, the hypothesis that individuals may have lived in rural zones, urban slums, or conflict zones of endemicity and/or migrated from them to other regions of the states cannot be ruled out (1,4).

A distinct male predilection was observed in our study, with a male-to-female ratio of 9.5:1. Other studies detected even higher proportions for males, as demonstrated in a Brazilian study in which all cases had occurred in males (24) and in a series from Argentina in which the male-to-female ratio was of 20:1 (12). In contrast, a previous study showed a similar distribution between men and women (ratio 1:1) (13). However, compelling evidence from 613 cases of oral PCM detected in single case reports and small case series showed marked male involvement in 90.2% of cases (7). The prevalence among males can be attributed to occupational (professional) issues, particularly in rural areas of Brazil, where male presence is very strong. Likewise, infections in men can be explained, in part, by a lack of the female hormone estrogen, which can inhibit the change of the saprobic mycelium form to the pathogenic yeast form of the fungus, preventing the development of PCM disease in women (25,26). In addition, males exhibit negative expression of estrogen receptor-alpha in oral PCM lesions (25). The clinicodemographic profile of women affected by oral PCM is quite similar to that of men, but some of them exhibit systemic changes such as HIV infection, depression, and pregnancy (26). Herein, about 60% of subjects were middle-aged and older adults, as also documented elsewhere (7-9). Conversely, in a study from Argentina, the mean age of affected individuals was 39 years (12). Other studies have also documented cases in pediatric populations (6,7,11), but this was not case in our analysis.

Clinically, oral lesions generally appear in single or multiple forms as “mulberry-like” ulcerations that most commonly affect the gingiva/alveolar ridge, palate, lips/labial commissure, and buccal mucosa (7), as also observed in our series. Studies have documented that the involvement of the gingival region can reach up to 78% of affected individuals (10,14). Previous findings are conflicting about the mechanism by which the fungus is directly inoculated into oral tissues in humans. Brazão-Silva *et al*. (6) suggested that inflammatory mediators produced in the context of pre-existing periodontitis could hypothetically contribute to the installation of the fungus. Yet, the contribution of gingival inflammation favoring the development of the disease after local inoculation of the fungus or only exacerbating the expression of the disease at the site is still a matter of debate (6). It is known that individuals can traumatize the oral mucosa by chewing vegeTable residues or using plant fragments as toothpicks, which consequently would act as a route of infection (6,9). On the other hand, in mice, the intraoral traumatic route did not seem to mimic the natural history of PCM (27).

Although in the present study clinicians listed PCM as the first diagnostic hypothesis, followed by oral squamous cell carcinoma (OSCC), this disease also stands out for its miscellaneous clinical manifestations. The list of differential diagnoses of oral PCM covers, but is not limited to, OSCC, oral histoplasmosis, oral leishmaniasis, acquired oral syphilis, oral tuberculosis, and oral lymphoma (6,7). Of note, although this is an exceedingly rare occurrence, previous reports have shown synchronous cases of oral PCM and OSCC (28). In our study, no concomitant conditions were noticed.

Currently, both international and Brazilian guidelines endorse that the mainstay of therapy for patients with mild­to­moderate clinical forms of the PCM is itraconazole (200 mg daily for 9-12 months), while amphotericin B is reserved for patients who are immunosuppressed (e.g., advanced HIV disease) (1,20). However, in the studies included in our review, several antifungals were reported to be effective in the treatment of different clinical forms of the disease, including azole derivatives, sulfonamide derivatives, and amphotericin B. Recently, experimental studies and case reports have revealed that photodynamic therapy can be an ancillary tool in the management of oral PCM, as it presents promising results in terms of accelerating the repair process and providing decontamination, progressively improving wound healing (29,30).

The limitations of the current study included the fact that data about the treatment and follow-up of affected individuals were unavailable, since the investigation involved only diagnostic data from an oral and maxillofacial diagnosis service. After the oral diagnosis, individuals are referred to hospitals to undergo treatment. Some other data were also not available in the registries, in particular risk factors (e.g., tobacco smoking, alcohol consumption, and HIV status) that are well established in the epidemiological aspects of PCM (1). Investigation of the lungs or other organs was not performed in this sample, which is also a shortcoming. In this context, we highlight that the notification of oral PCM in Brazil is important for the implementation of preventive measures and early diagnoses that can lead to more effective treatments and fewer sequelae for this population.

In summary, this study confirms the epidemiology of oral PCM as an outcome with a marked predilection for middle-aged and older male adults. Clinicians should be aware of the oral manifestations of PCM, recapitulating the clinicodemographic aspects and differential diagnoses and not limiting the definition of the disease as lesions with a granulomatous appearance. In suspected cases, the clinician should thoroughly investigate the patients' habits, as well as whether they live or work in endemic or hyperendemic regions, mainly rural and/or urban zones.
